# Insights in Pt-based electrocatalysts on carbon supports for electro-oxidation of carbohydrates: an EIS-DEMS analysis

**DOI:** 10.3389/fchem.2024.1383443

**Published:** 2024-05-09

**Authors:** Eleazar Castañeda-Morales, Fabio A. Gómez-Gómez, Yueyin Li, Arturo Manzo-Robledo

**Affiliations:** ^1^ Instituto Politécnico Nacional, Laboratorio de electroquímica y corrosión. Escuela Superior de Ingeniería Química e Industrias Extractivas, Av. Instituto Politécnico Nacional S/N, Unidad Profesional Adolfo López Mateos, Mexico City, Mexico; ^2^ Universidad de Anahuac Campus norte, Mexico City, Mexico

**Keywords:** fuel cells, glucose oxidation reaction, Pt catalyst, carbon-based support-materials, nanoparticles, DEMS

## Abstract

In this work, the electrochemical oxidation of carbohydrates (glucose, fructose, and sucrose) was induced at the interface of Pt-nanoparticles supported on different carbon-based materials as carbon vulcan (C) and carbon black (CB). It was found that the support plays an important role during carbohydrates electro-oxidation as demonstrated by electrochemical techniques. In this context, current-concentration profiles of the redox peaks show the behavior of the pathways at carbohydrates-based solutions. Herein, the trend of current measured was glucose > sucrose > fructose, attributed to differences in the organic functional groups and chain-structure. Raman, XRD, SEM-EDS and XPS put in clear important structural, morphological, and electronic differences linked with the intrinsic nature of the obtained material. Differential Electrochemical Mass Spectroscopy (DEMS) indicated that the selectivity and the conversion of the formed reaction products during oxidation is linked with the catalyst nature (distribution, particle size) and the interaction with the carbon-based support.

## 1 Introduction

Carbohydrates can be regarded as the most abundant compounds, making up 80% of the biomass in the world. The structural composition of carbohydrates depends largely on saccharides, which can be mono-, disaccharides, dextrins, or oligosaccharides. Glucose, fructose, and sucrose, among other carbohydrates can be used as energy sources for a variety of organisms, from bacteria to humans. Additionally, these carbohydrates find widespread use in the preparation of food and beverages, employed as sweeteners (Torto, 2009). The electrochemical oxidation of carbohydrates opens up possibilities for various applications, including the development of direct fuel cells, cardiac pacemakers, and the creation of a glucose sensor for use in conjunction with an artificial pancreas (Kelaidopoulou et al., 1999). With the objective of developing new energy sources, carbohydrates are utilized as biofuel due to their easy availability, non-toxic nature, and lack of storage or explosion-related issues, in contrast to systems such as hydrogen fuel cells. On the other hand, the oxidation of glucose to carbon dioxide yields very high energy (−2.87 × 10^6^ J/mol), with the exchange of 24 electrons (Basu and Basu, 2010).

Generally, carbohydrates consumption in a fuel cell can be divided based on the type of catalyst employed to facilitate electrode reactions, such as enzymatic or non-enzymatic catalysts. Enzymatic bio-fuel cell operations (EBFC) are complex, resulting in lower power and current output. These devices present low-term stability and demand high maintenance for the immobilization of enzymes on the electrode surface (Brouzgou and Tsiakaras, 2015). To overcome the complexity and drawbacks associated with enzymatic bio-fuel cells, direct electro-oxidation of carbohydrates on non-enzymatic noble metal electrodes can be employed (Basu and Basu, 2010). Studies conducted over the last few decades indicate that noble metal catalysts, particularly those based on polycrystalline platinum can electro-oxidize glucose, fructose, and sucrose with high performance. These studies have identified different reaction mechanisms in an acidic medium (Kelaidopoulou et al., 1999).

In the case of electrochemical oxidation of glucose on platinum as a catalyst, the products during reaction could be organic compounds such as gluconolactone and gluconic acid, among others, which can be regarded as value-added products (Basu and Basu, 2012; Chen et al., 2015; Faverge et al., 2023). Throughout the various paths of glucose electrooxidation, different amounts of intermediate molecules are produced, depending on the applied potential (Basu and Basu, 2010; Brouzgou and Tsiakaras, 2015). These species strongly adsorb to the electrode surface, dropping electrode performance due to decreased active sites. Decomposition of these organic molecules and their desorption products from the surface occur at high positive potentials (Kelaidopoulou et al., 1999). On the other hand, the carbon-based catalyst support, such as carbon vulcan or carbon black, plays a crucial role in modulating electro-oxidation processes. The use of carbon-based materials as catalytic supports allows the increases the number of active sites during adsorption/desorption processes. It has been demonstrated that the crystalline structure and point of zero charge (pzc) of the carbon supports play an important role in the electrocatalytic activity. Previously, PtRu nanoparticles have been supported on carbon material and a relation between the disorder-carbons degree and catalytic performance was found at more crystalline materials such as graphene and carbon nanotubes, showing a higher activity in the hydrogen oxidation reaction (HOR), Hasa et al., 2019. However, studies with amorphous carbons as catalytic supports for electro-oxidation of complex organic molecules (i.e., glucose, fructose, and sucrose) still needs to be investigated. The influence of carbon-support can be discussed by two approaches: i) the extent of over-oxidation might depend on the internal rate of diffusion, and ii) the support might influence the surface properties of platinum particles due to metal-support interactions (Vleeming et al., 1997).

On the other hand, the use of *in-situ* techniques during operation is particularly useful to reveal reaction pathways evaluating the selectivity of the catalyst and to monitoring the formed species. Differential Electrochemical Mass Spectrometry (DEMS) has been employed as an efficient tool to identify gaseous and volatile reaction products in processes such as the electrocatalytic reduction of CO_2_ (Mora-Hernandez et al., 2021b) or hydrogen production (González-Anota et al., 2023), as well as during the oxidation of organic compounds. DEMS technique implies an electrochemical reactor coupled to a mass spectrometer which allow the identification and quantification of reaction intermediates and products through mass to charge ratio (m/z). In the case of the electrooxidation of carbohydrates, in specific glucose, DEMS allows monitoring the detection of some reaction products as well as the generation of oxygen (m/z = 32) and carbon dioxide (m/z = 44).

In this work, the electrocatalytic properties of platinum (Pt) nanoparticles supported on carbon vulcan (C) or carbon black (CB) with a metal loading of 10% (nominal), synthesized through the impregnation methodology for the electrooxidation of glucose, fructose, and sucrose are reported. The preparation and physical characterization of various electro-catalysts using scanning electron microscopy (SEM), Raman spectroscopy, and X-ray diffraction (XRD) are also reported. Glucose, fructose, and sucrose electrooxidation on Pt/C and Pt/CB catalysts in an acid medium are electrochemically characterized using cyclic voltammetry (CV) and electrochemical impedance spectroscopy (EIS) techniques. Finally, it was demonstrated that the effect of the support (C and CB) in the platinum electrocatalyst influences the activity towards the electrochemical oxidation reaction of glucose. This impact is observed on mass-transport and charge-transport limitations during anodic polarization, as demonstrated using the *in-situ* mass spectroscopy (DEMS) technique.

## 2 Experimental section

### 2.1 Materials and reagents

Sodium hexachloroplatinate (IV) hexahydrate (Na_2_PtCl_6_.6H_2_O, Sigma-Aldrich, 98%), sodium borohydride (NaBH_4_, Sigma-Aldrich, 99.99%), Carbon vulcan (XC-72R, Fuel cell store), carbon black (pigment powder N330), Methanol (CH_3_OH, Meyer, ≥99.8%), and deionized water (DI, Fermont) were employed for synthesis. Whereas, D-(+)-glucose (C_6_H_12_O_6_, Sigma-Aldrich, ≥99.5%), D-(+)-fructose (C_6_H_12_O_6_, Sigma-Aldrich, ≥99%), and D-(+)-sucrose (C_12_H_22_O_11_, Merck, 99.9%) were used as a probe molecules. Sulfuric acid (H_2_SO_4_, Fermont, 97.2%) were used as supporting electrolyte. All chemicals were used without further purification.

### 2.2 Synthesis of Pt/C and Pt/CB

The procedure was made following the impregnation methodology (Delannoy et al., 2006). The preparation of platinum nanoparticles supported on carbon structures (carbon vulcan, C; or carbon black, CB) started by preparing a suspension of carbon support in CH_3_OH. Then, an aqueous solution of sodium hexachloroplatinate hexahydrate, Na_2_PtCl_6_.6H_2_O as metallic precursor of platinum is added with the carbon suspension. The mixture was stirred at 65 C for 1 h in an argon atmosphere. Afterward, NaBH_4_, as reducing agent, was added to the suspension. The suspension was maintained under stirring for 2 h. Finally, the obtained powder was dried at 100°C. The samples Pt/Carbon Vulcan and Pt/Carbon back were denoted as Pt/C and Pt/CB with a metal:support ratio of 1:9.

### 2.3 Structural and morphological characterization

The phase composition of the samples (Pt/C and Pt/CB and their corresponding catalytic supports) were studied by X-ray diffraction (XRD) using a Bruker D2 Phaser second Gen X-ray diffractometer equipped with Cu-Kα radiation source (1.5406 Å) operated at 40 keV, and 15 mA in a 2θ range from 10° to 90°. The morphological characteristics of the samples (Pt/C and Pt/CB) were studied using a scanning electron microscope (SEM). The SEM images were obtained on a JEOL JSM 6701F microscope with a secondary electron detector at an acceleration of 60 kV. The SEM is equipped with an energy-dispersive X-ray spectroscopy (EDS) and was used to obtain the chemical elemental analysis. Structural characteristics of the carbon-based catalytic supports were evaluated using Raman spectroscopy at a shift range from 500 to 2,300 cm^−1^ on an i-Raman Plus B&W Tek Micro Raman spectrometer with an excitation wavelength of 785 nm. The electronic properties and oxidation states of the synthesized platinum nanoparticles supported on carbon-based materials (carbon vulcan and carbon black) were investigated from X-ray photoelectron spectroscopy (XPS). The analysis was performed using a K-alpha Thermo Fischer Scientific spectrometer with a monochromatic Al Kα source (1486.6 eV). The X-rays were micro-focused at the source to give a spot size on the sample of 400 mm in diameter. Samples remained under vacuum for more than 10 h in a pre-chamber directly connected to the equipment and then transferred to the analysis chamber-zone with a base pressure of 1 × 10^−9^ Torr that remained constant throughout the experiment.

### 2.4 Electrochemical characterization

All electrochemical measurements were carried out using a potentiostat-galvanostat (Autolab PSGSTAT-302) in a three-electrode cell setup. A carbon rod and a standard hydrogen electrode (SHE, containing 0.5 M H_2_SO_4_) were used as the counter electrode (CE) and as the reference electrode (RE), respectively. Glassy carbon disk (GC, 3 mm in diameter) was used as the working electrode (WE). The GC electrode was polished to a mirror-finished surface using alumina powder (ca. 0.3 µm). Homogeneous catalyst ink was prepared by dispersing a mixture of 5 mg of catalyst in 1 mL water-isopropanol solution (volume ratio 1:1) and 70 µL of 5 wt% Nafion in an ultrasonic treatment for 30 min. Thereafter, the GC electrode was coated with 5 µL of the obtained suspension and dried at room temperature. The total catalytic mass loading was 0.33 mg_catalyst_/cm^2^.

Cyclic voltammetry (CV) was used for the electrochemical characterization to evaluate electrocatalytic activity in the potential window from 0.05 to 1.2 V vs. SHE with a scan rate of 5 mV/s. The capacitances were obtained by recording CVs in a potential window of 0.35–0.45 V vs. RHE (region with non-faradaic current) at different scan rates in the range from 75 to 500 mV/s. To determine the electrochemically active surface area (ECSA), cyclic voltammetry in the presence of 1 M KCl and 10 mM $K_{3}\text{Fe}{\left(\text{CN}\right)}_{6}$ was employed with scan rates between 5 and 100 mV/s. Electrochemical impedance spectroscopy (EIS) was carried out in potentiostatic mode applying a sinusoidal potential signal in the frequency interval from 100,000–0.1 hz using an amplitude of 10 mV. An aqueous Ar-saturated 0.5 M H_2_SO_4_ solution was used as supporting electrolyte in the absence or presence of 0.5 M of the corresponding carbohydrate (glucose, fructose, or sucrose). EIS data analysis was carried out using Zview software by fitting the data to an equivalent circuit.

### 2.5 Differential electrochemical mass spectrometry (DEMS) experimentation

A home-made three-electrode configuration DEMS cell was used for recording the ionic and faradic currents as a function of electrode potential (and time). The electrochemical measurements were performed using a potentiostat-galvanostat (µAutolab III). The cell components (WE, CE, RE and electrolyte) employed for DEMS studies were made as the description for *ex-situ* analysis in Section 2.4. The ionic current (mass signal) for selected mass-to-charge ratios (m/z) were recorded simultaneously with respect to the faradaic current-potential profiles at a scan rate of 1 mV/s. The experiments were carried out employing H_2_SO_4_ 0.5 M solution as electrolyte in absence and in presence of carbohydrates. The electrochemical cell was connected to the quadrupole mass spectrometer (Prisma QMG220) at a working pressure of ca. 2.0 × 10^−5^ mbar. All solutions were saturated with argon prior to the DEMS experimentation.

## 3 Results and discussions

### 3.1 Structural characterization

**FIGURE 1:**
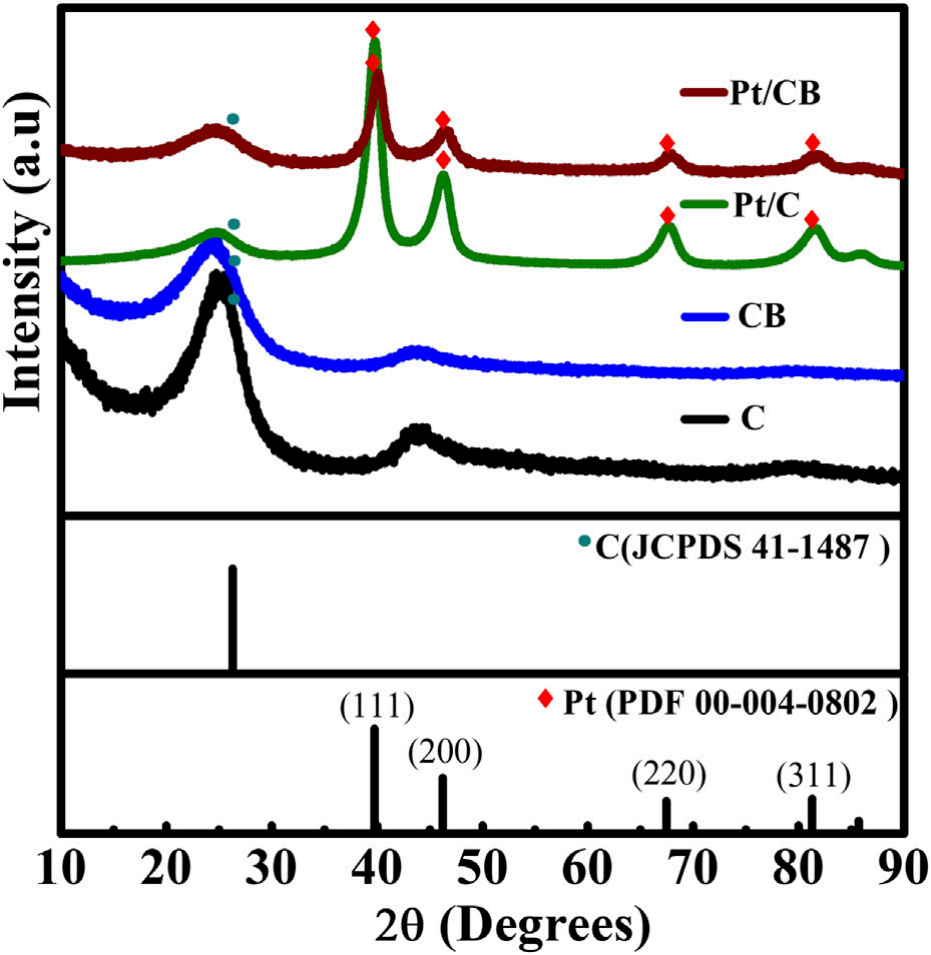
DRX patterns of the materials of C, CB, Pt/C and Pt/CB.

X-ray diffraction was employed to identify phases, determine crystallite size, and to observe changes in lattice parameters of the prepared catalysts and their corresponding supports: carbon vulcan (C) and carbon black (CB); as shown in Figure 1. The diffraction peaks of the powders at 2θ = 39.76^o^, 46.24^o^, 67.45^o^ and 81.289^o^ correspond to the (111), (200), (220) and (311) planes, respectively; resulting in a Platinum face-centered cubic polycrystalline systems according to (JCPDS 00−004−0802). The diffraction peaks between 26.3^o^ (2θ) are assigned to carbon Vulcan and carbon black supports (JCPDS# 00-41-1487) (Pérez-Sosa et al., 2021). The average crystallite size was obtained with Scherrer model, given by Equation (1), using the main-platinum peak corresponding to the (111) plane in Figure 1 (Basak et al., 2022).

$$d=\frac{K*\lambda }{B*\cos\theta }$$ (1)

The crystallite size (d) is determined by utilizing X-ray diffraction, where the shape factor (K) ranges from 0.89 to 1.39. Other variables include the wavelength of X-ray radiation (λ), the diffraction angle position (θ), and the full width at half maximum (FWHM) of the diffraction peak at the 2θ angle (in radians, denoted as B). The synthesis method used produced platinum crystallite size (nm) in the nanometric scale, in the values found to be 6.32 nm for Pt/C and 6.27 nm for Pt/CB.

**FIGURE 2:**
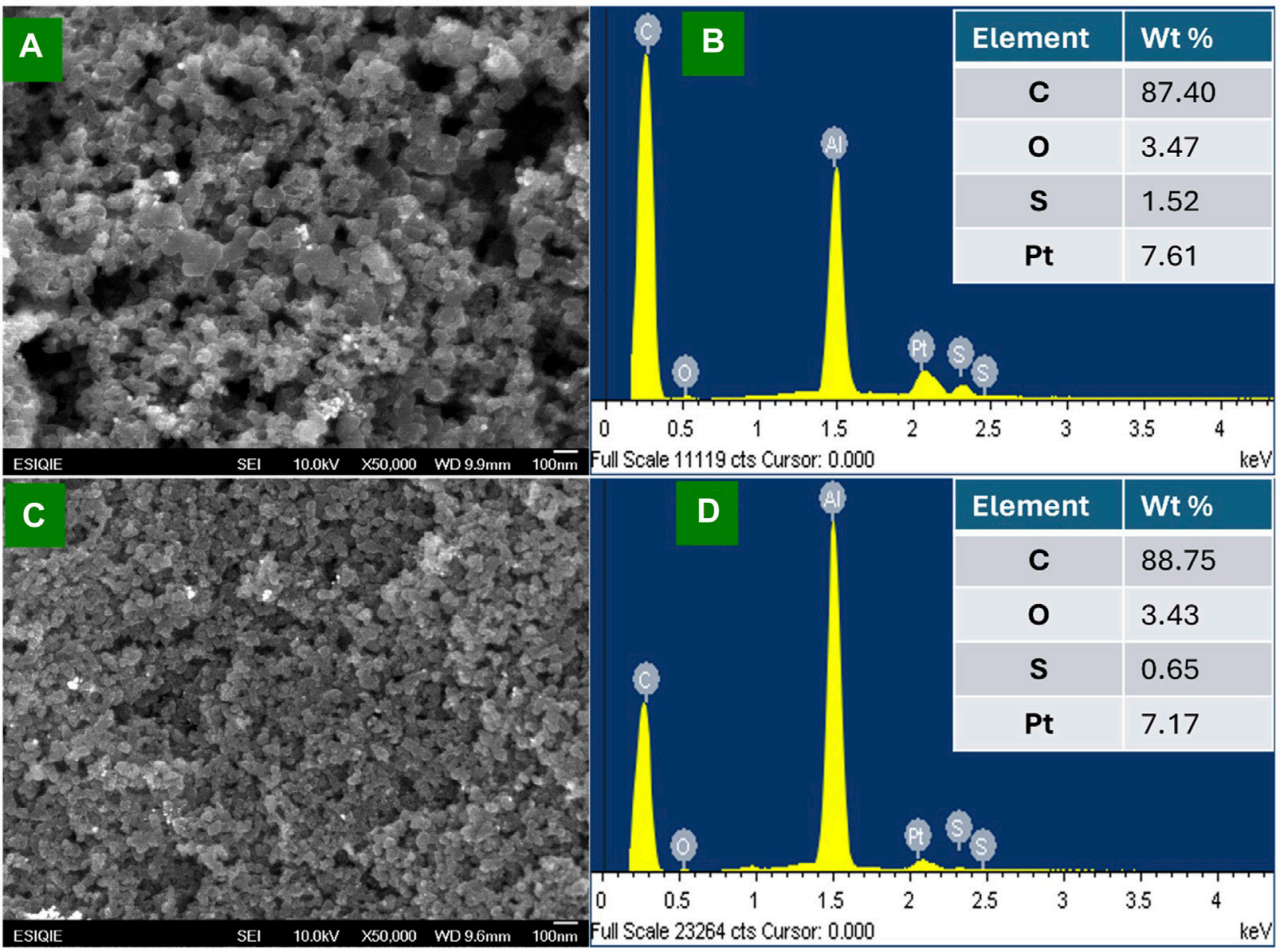
SEM images and their corresponding spectrum of energy dispersive spectroscopy (EDS) of the electrocatalysts **(A, B)** Pt/C and **(C, D)** Pt/CB.

The morphological characteristics and the chemical analysis of the synthetized Pt/C and Pt/CB catalysts were studied by SEM-EDS. Figure 2 shows the micrographs for **(a)** Pt/C and **(b)** Pt/CB. Semi-spherical carbon nanoparticles and the well-dispersed Pt nanoparticles can be observed on the carbon surfaces from the SEM images. The EDS analysis, seen in Figure 2 for **(b)** Pt/C and **(d)** Pt/CB, verified the presence of C, O (corresponding to carbon support) and Pt (corresponding to metallic nanoparticles). According with the results, it could be noticed that the catalysts exhibited the similar contents of oxygen, carbon, and platinum.

**FIGURE 3:**
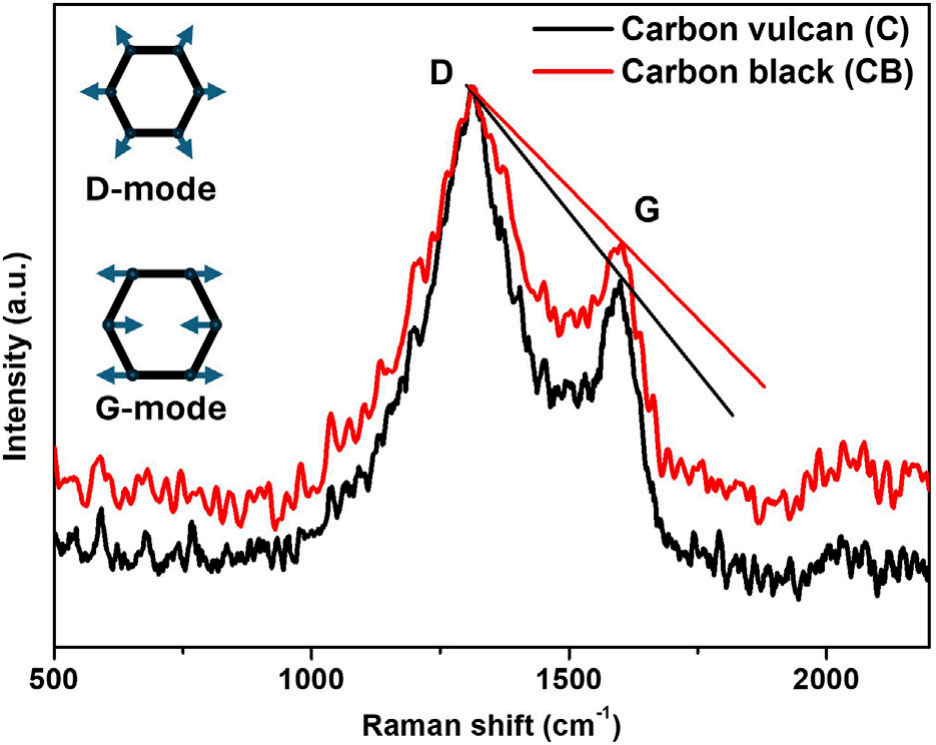
Raman spectra of the carbon-based materials carbon vulcan (C) and carbon black (CB).

On the other hand, Raman spectroscopy was used to characterize changes in the structure as well as the degree of defectiveness of the carbon materials (carbon Vulcan and carbon black). In Figure 3, the displayed Raman spectra exhibited a slight overlapping of D and G band peaks at ca. 1313 cm^−1^ and 1600 cm^−1^ respectively for both materials. The D-band is assigned to structural disorder in graphite lattice in the A_1g_ breathing mode of the 6-fold aromatic rings near the basal edge. The G-band is assigned to the crystallinity in graphite lattice by hexagonal carbon structure, associated with the E_2g_ vibration mode (Wu et al., 2018; Lee et al., 2021). The ratio of D-peak to the G-peak intensity denoted as (I_D_/I_G_) indicates the degree of defective carbon structure (Zhang et al., 2002). The values of I_D_/I_G_ for the studied materials were found to be 1.31 and 1.23 for carbon Vulcan and carbon black respectively, suggesting that carbon Vulcan shows a higher amount of disorder compared to the other carbon-based material. In this context, Raman spectroscopy was further used to determinate the crystallite size (L_a_), obtained from the ratio I_D_/I_G_ using the model given by Equation (2) (Guerrero-Contreras and Caballero-Briones, 2015).

$$L_{a}\left(\text{nm}\right)=\frac{560}{E_{l}^{4}}{\left(\frac{I_{D}}{I_{G}}\right)}^{-1}$$ (2)

Where E_l_ is the energy laser excitation used in the experiment. The obtain values for the crystallite size (L_a_) were found to be 69.5 and 74.1 nm for carbon Vulcan and carbon black respectively. This decrease in crystallite size by carbon vulcan can be attributed to the diminution in the graphitic domains due to structural disorder.

**FIGURE 4:**
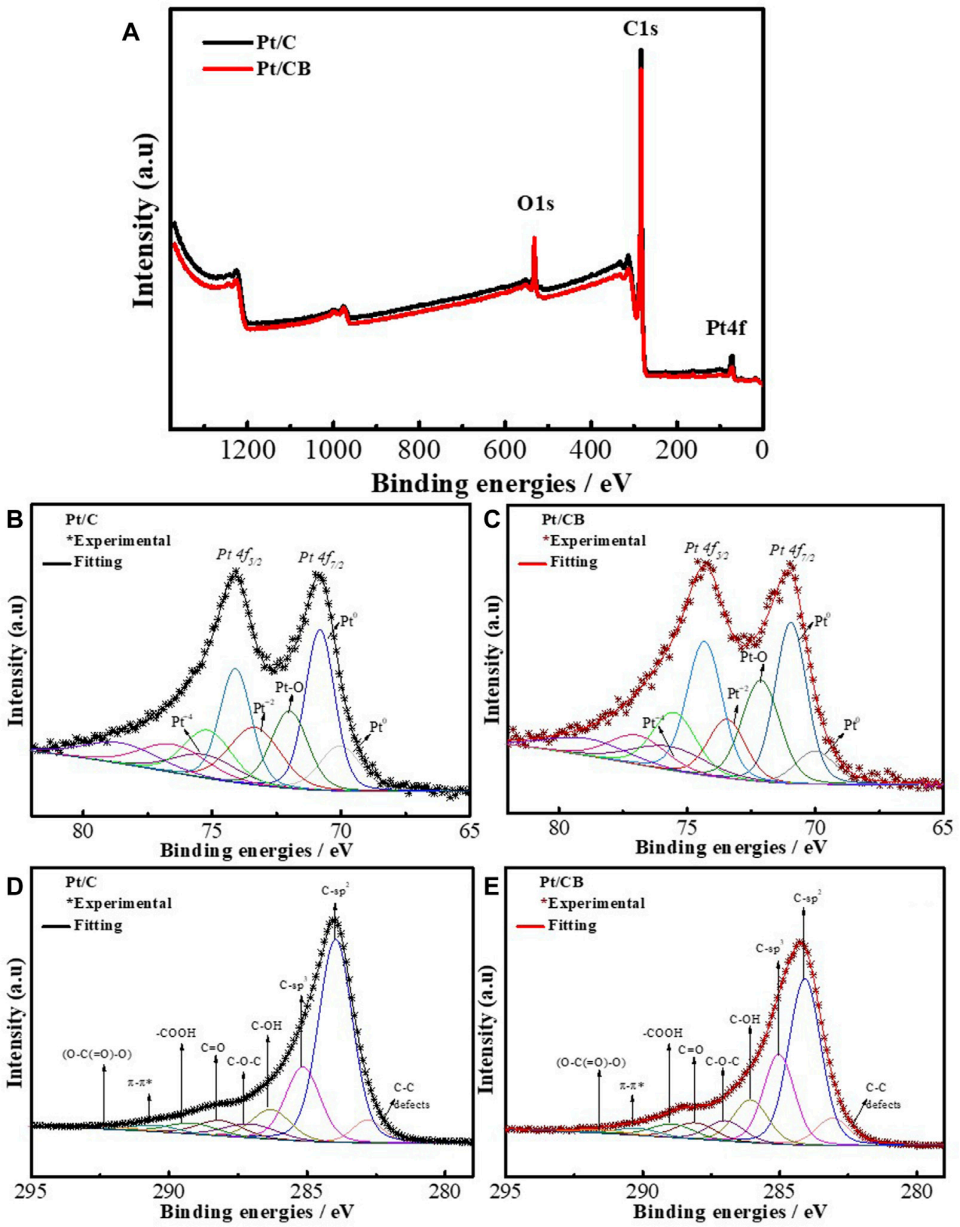
**(A)** XPS wide spectra of Pt/C and PtNi/C, where main regions for C1s, O1s and Pt4f are indicated. High-resolution XPS spectra for Pt4f obtained for Pt/C **(B)** and Pt/CB **(C)** catalysts. High-resolution XPS spectra and deconvolution for the main C1s found for Pt/C **(D)** and Pt/CB **(E)**.

To acquire more insights into the electronic and chemical state of the surface of the samples, photoelectron X-ray spectroscopy was carried out. Figure 4A shows the XPS wide scans for the Pt/C and Pt/CB samples, where main regions for C1s (284.08 ± 0.2 eV), O1s (532.08 ± 0.2 eV), Pt4f (71.08 ± 0.2 eV) can be observed. Figure 4B–C show high-resolution XPS spectra for Pt4f acquired from Pt/C and Pt/CB catalysts. These spectra were fitted with the principal Pt4f_7/2_ and Pt4f_5/2_ peaks for both materials. Spectra were deconvoluted in distinct Pt components: Pt^0^ and Pt-O. Additionally, the third and fourth contributions of Pt^+2^ and Pt^+4^ are associated with PtCl_2_ and PtCl_4_, respectively, traces from the precursor Finally, an individual contribution is observed for Pt5p_1/2_, completing the spectral analysis for platinum species (Guerrero-Ortega et al., 2018). A significant difference in the electronic states of platinum in the samples was not observed, indicating that there was not a substantial effect of the carbon supports towards the formed platinum nanoparticles performance.

The high-resolution XPS spectrum displayed in Figure 4D–E reveals differences within the intrinsic structure of both catalytic supports. the C1s spectra exhibit contributions from diverse chemical species and functional groups. The energy of the main peak for both samples is 283.9 ± 0.2 eV and corresponds to carbon with the graphite structure (sp2 hybridization, C=C). Table 1 organizes the adjusted binding energies and atomic compositions (in %) of the C1s spectra, which were derived from various chemical species and functional groups presented in the analyzed samples. The presence of C-C signals suggests the existence of defect zones possibly from carbon-active vacancies. Conversely, signals indicative of carbon sp2 hybridization were detected on the surface or within layers. Meanwhile, signals attributed to C-sp3 hybridization, along with bonds associated with species such as C-OH, C-O-C, C-O, COOHO, π-π *, and (O-C(O)-O), are also identified. These contributions correspond to signals assigned to a various functional groups on the catalyst surface which is associated to modulations in adsorption/desorption processes of reactants (Fujimoto et al., 2016).

**TABLE 1:** Binding energy (eV) and weight percentage of total area of Pt5p, Pt4f7/2, Pt4f5/2, and Cs1 from XPS analysis.

Catalyst		Pt 5p	Pt 4f_7/2_				Pt 4f_5/2_				At%			
		**Pt** ^**0**^	**Pt** ^**0**^	**Pt-O**	**Pt** ^**+2**^	**Pt** ^**+4**^	**Pt** ^**0**^	**Pt-O**	**Pt** ^**+2**^	**Pt** ^**+4**^	**Pt** ^**0**^	**Pt-O**	**Pt** ^**+2**^	**Pt** ^**+4**^
Pt/C	BE[+−0.2 eV]	70.02	70.82	72.00	73.34	75.32	74.10	75.18	76.51	78.56	43.83	22.09	20.86	13.23
Pt/CB	BE[+-0.2eV]	70.00	70.95	72.12	73.44	75.70	74.32	75.50	76.94	78.88	45.33	26.03	15.76	12.89
		**C-C**	**C-sp** ^**2**^	**C-sp** ^**3**^	**C-OH**	**C-O**	**C=O**	**-COOH**	π–π*	**(O-C(=O)-O)**				
Pt/C	BE[+−0.2 eV]	282.79	283.97	285.15	286.3	287.05	288.15	289.16	290.51	291.95				
	At%	4.92	50.56	18.73	8.33	5.3	5.1	3.98	2.16	0.92				
Pt/CB	BE[+−0.2 eV]	283.05	284.08	285.03	286.06	286.89	288.09	288.91	290.17	291.39				
	At%	6.7	41.42	21.59	11.39	5.81	5.04	4.49	2.32	1.23				

### 3.2 Electrocatalytic performance

In this section, the activity of the Pt/C and Pt/CB in terms of the influence of carbon catalytic-support functionalities by the observed current-versus-potential characteristics is discussed. Subsequently, the electrooxidation of carbohydrates (Glucose, Fructose and Sucrose) is evaluated in terms of their voltametric features and the mass-charge transport properties-surface-interactions. Finally, the corresponding characterization of generated products from glucose oxidation reaction, investigated by DEMS, is discussed.

#### 3.2.1 Electrocatalytic characterization of platinum on carbon supports

**FIGURE 5:**
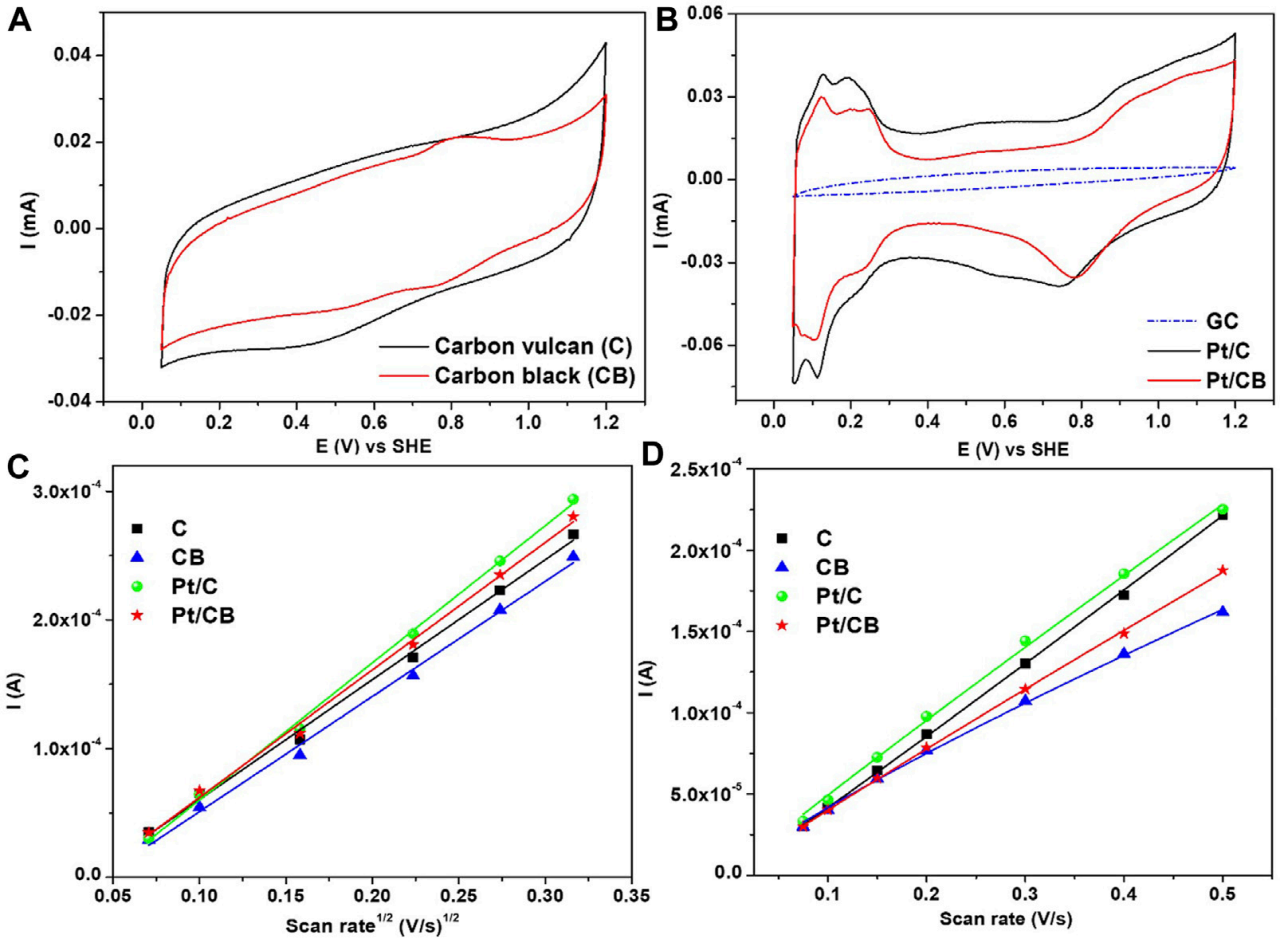
Current-potential profiles of **(A)** catalytic supports (carbon vulcan and carbon black) and their corresponding **(B)** Pt-based catalysts recorded in Ar-saturated 0.5 M H_2_SO_4_. Scan rate of 50 mV/s **(C)** Current vs. scan rate^1/2^ profiles of the samples (catalytic supports and Pt-containing) in 1 M KCl solution containing 10 mM K_3_Fe(CN)_6_ for the determination of ECSA. **(D)** Current vs. scan rate profiles of the samples (catalytic supports and Pt-containing) in 0.5 M H_2_SO_4_ solution for the determination of double layer capacitance.

Electrochemical studies of the catalysts were performed using a solution containing 0.5 M H_2_SO_4_ at a scan rate of 50 mV/s previously saturated with argon to avoid interferences from dissolved gases (i.e., oxygen). The current response started from the open-circuit potential (OCP) towards positive-going scan. In Figure 5A, the current-potential profiles for the catalytic supports are shown. The current response was performed in a potential interval from 0.05 to 1.2 V vs. SHE, at room temperature. It is important to notice that the voltametric features exhibited a typical behavior of carbon-based materials without remarkable redox peaks (Bose et al., 2022). Carbon Vulcan showed a higher capacitive current compared to carbon black. Figure 5B shows the current-potential profiles for the Pt-based catalysts. The glassy carbon characteristic was included for comparison. Both catalysts (Pt/C and Pt/CB) displayed the three potential-characteristic regions of a polycrystalline Pt structure where could be seen i) the desorption/adsorption region of hydrogen from Pt, ii) the region of the electric double layer and iii) the formation of oxides (PtO_x_) with the corresponding reduction towards metallic Pt (Liu et al., 2007). Therefore, the voltametric characteristics verify the presence of Pt on the carbon matrix as demonstrated by XRD and SEM-EDS. According to these results, the Pt on carbon Vulcan material (Pt/C) exhibited a similar response as carbon-supports analysis (see Figure 5A), where a higher capacitive current was obtained.

To a further analysis of electrocatalytical properties, the electrochemical surface area (ECSA) was investigated. This active area is an important parameter to analyze the electrocatalytic-intrinsic performance; cyclic voltammograms were collected at different scan rates in 1 M KCl and 10 mM $K_{3}\text{Fe}{\left(\text{CN}\right)}_{6}$ , for ECSA calculation. The response of current as a function of the square root of the scan rate is presented in Figure 5C. The obtained results show a relation given by Randles-Sevcik model (Equation 3) (Paixão, 2020).

$$i_{p}=2.69x10^{5}A\;C\;D^{1/2}\;n^{3/2}\;\upsilon^{1/2}$$ (3)

Where ${\;i}_{p}$ is the peak current, the value $2.69x10^{5}$ is a physical constant derived from the Randles-Sevcik model in the units of C/mol ; A is the electrochemical surface area, C is the concentration of redox species $K_{3}\text{Fe}{\left(\text{CN}\right)}_{6}$ , D is the diffusion coefficient ( $7.6\;x10^{-6}\;{\text{cm}}^{2}/s$ ), n is the number of transferred electrons and υ is the scan rate. The collected data was fitted with a linear model to determine the slope employed in Randles-Sevcik equation as given by Equation 4.

Y=a+bX (4)

where Y is the peak current of the redox couple ( $i_{p}$ ), *b* is the slope ( $i_{p}/\upsilon^{1/2}$ ), *X* is the square root of scan rate ( $\upsilon^{1/2}$ ), and *a* is the intercept to the y-axis. The slope in this equation is linked to capacitive current of the material and can be employed to determine the ECSA given by the term (A) in Equation 3. To validate the capacitive properties, the double layer capacitance $\left(C_{\text{dl}}\right)$ was determined by cyclic voltammetry collected at different scan rates (75–500 mV/s) according to a previously reported procedure (Han et al., 2015). For ideal capacitive systems, the $C_{\text{dl}}$ is given by Equation 5.

$$i_{C}=\upsilon \;C_{dl}$$ (5)

where ${\;i}_{C}$ and υ are denoted by the charging current and the scan rate, respectively. The measurements were made in 0.5 M H_2_SO_4_ solution to estimate the double layer capacitance of the samples. Data was fitted with a linear model for regression of the variables Y and X (see Figure 5D), as described in Equation 6.

$$Y=bX^{\alpha }$$ (6)

where *Y* is the capacitive current, *b* is the capacitance, *X* is the scan rate and the exponent α is related with the reversibility of the reaction. The obtained data were fitted with an allometric regression to assess deviations from linearity; at α=1 , the electrical interaction can be interpreted as an ideal capacitor behavior as described in Equation (5). Based on the findings, carbon vulcan-based materials (with and without platinum nanoparticles) show a higher capacitance, which is related with a more disordered carbons with smaller-graphene domains that leads to higher capacitances (more storage of ions on the surface) (Liu et al., 2023). The obtained ECSA and $C_{\text{dl}}$ values were compared in Table 2.

**TABLE 2:** Electrochemically active surface area (ECSA) and double layer capacitance (C_dl_) of the materials in study.

Sample	ECSA [cm^2^/mg_catalyst_]	C_dl_ [µF]
Carbon Vulcan (C)	5.37	441.4, α = 0.958
Carbon black (CB)	5.163	294.7, α = 0.849
Pt/C	6.175	454.7, α = 1.038
Pt/CB	5.818	361.4, α = 0.954

#### 3.2.2 Activity towards the electrooxidation of carbohydrates

**FIGURE 6:**
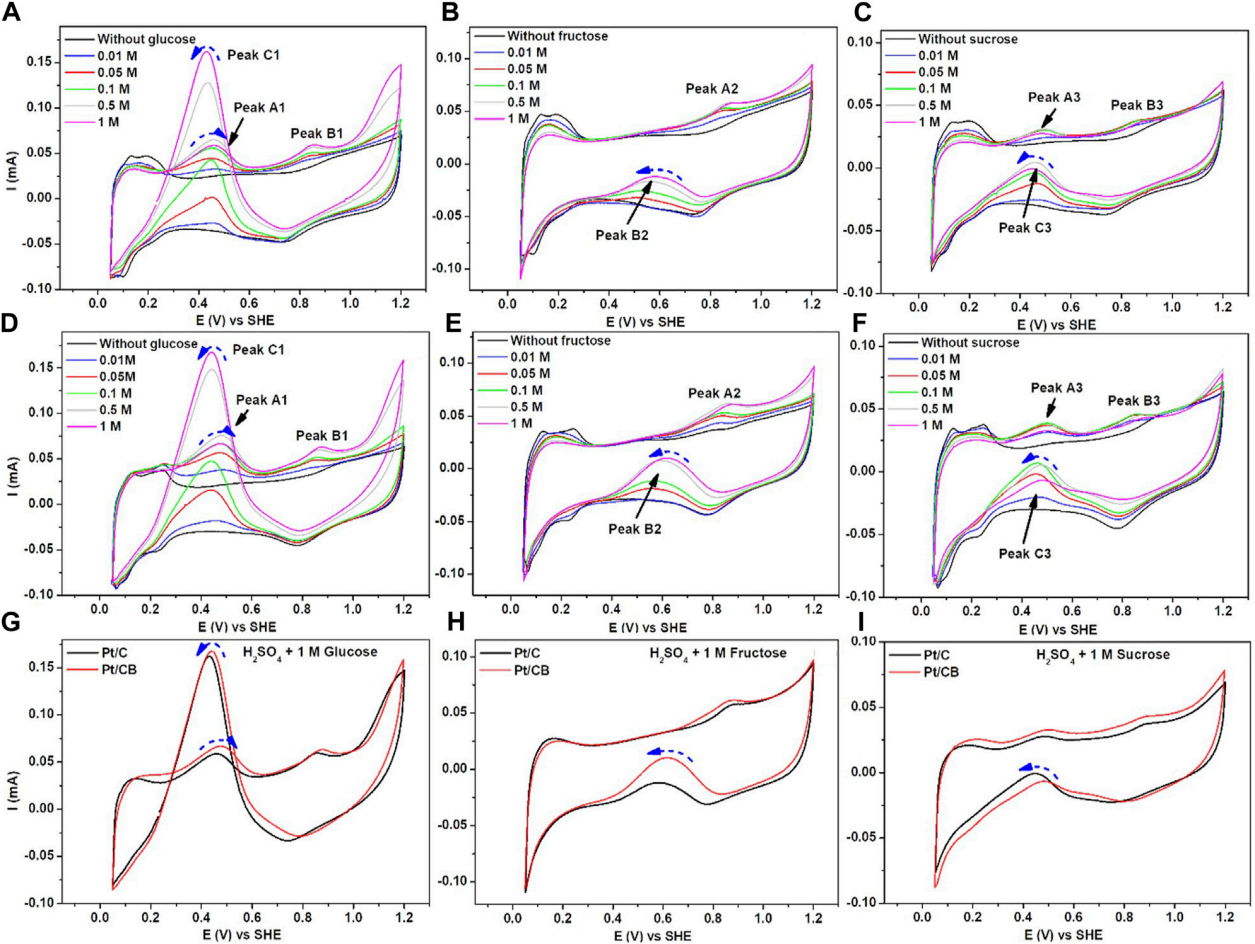
Current-Potential profiles of **(A–C)** Pt/C and **(D–F)** Pt/CB in electrooxidation of glucose, fructose and sucrose at different concentrations using 0.5 M H_2_SO_4_ as supporting electrolyte. Scan rate of 50 mV/s. Comparison between Pt/C and Pt/CB in a solution of 0.5 M H_2_SO_4_ in the presence of 1 M of **(G)** glucose, **(H)** fructose and **(I)** sucrose.

To compare the electrocatalytic activity of Pt/C and Pt/CB towards the electrooxidation of the three carbohydrates in this study, cyclic voltammetry was used, as shown in Figure 6. The voltametric features were recorded in a solution containing 0.5 M H_2_SO_4_, both free-analyte and in the presence of different concentrations of the carbohydrate in turn, in this case, **(a,d)** glucose, **(b,d)** fructose and **(c,f)** sucrose. It is important to remark that the electro-oxidation of the carbohydrates are a complex process influenced by the reaction conditions (pH, temperature), supporting electrolyte and the surface structure of the catalyst (Yang et al., 2015), and the precise reaction pathways in the different potential regions are not totally identified. Considering an electrochemical arrangement, based in a proton exchange membrane (PEM), the reactions that take place are given by equations 7 and (8) for the anode and the cathode respectively (Kerzenmacher et al., 2008b).

$$C_{6}H_{12}O_{6}+6H_{2}O\to 6{\text{CO}}_{2}+24H^{+}+24e^{-}$$ (7)

$$6O_{2}+24H^{+}+24e^{-}\to 12H_{2}O$$ (8)

Nevertheless, CO_2_ is one of several products (such as organic molecules with lower molecular weight) that can be generated during glucose reaction, indicating that the molecule is not fully oxidized (Faverge et al., 2023). In the past, reaction pathways have been proposed to understand the oxidation of this molecule towards several organic products. In this context, gluconic acid is considered one of the main products in acid and neutral conditions (Kerzenmacher et al., 2008a), as given in Equation 9.

$$C_{6}H_{12}O_{6}+H_{2}O\to C_{6}H_{12}O_{7}+2H^{+}+2e^{-}$$ (9)

When gluconic acid is formed, it is re-oxidized to form other organic intermediates. However, these reactions rates are slower than glucose oxidation. According with the literature (Skou, 1977; Abbadi and van Bekkum, 1995; Kerzenmacher et al., 2008a), a general oxidation from electrochemical pathways could be i) adsorption of glucose molecule on the Pt surface, ii) Oxidation towards gluconic acid, by the cleavage of the aldehyde group, iii) Oxidation towards organic intermediates, among them 2-ketogluconic acid, glycol aldehyde, and 2-hidroxy-3-keto-4-pentaoic acid, iv) formation of organic compounds with lower molecular weight such as acetaldehyde, formic acid along with CO_2_. For this study, the glucose oxidation reaction was performed, as seen in Figure 6 for **(a)** Pt/C and **(d)** Pt/CB catalysts. As observed, three redox peaks are identified, denoted as peak A1, peak B1 and peak C1 corresponding to three oxidation mechanisms in the organic molecule. Remarkably, high oxidation current was observed for peak C1 in the negative-going scan, which could be attributed to a strong desorption process of products. The current response is modulated by the concentration of glucose. On the other hand, fructose was oxidized in the presence of the Pt-based catalysts, displayed in Figure 6 (b) Pt/C and **(e)** Pt/CB. In the presence of this carbohydrate, two oxidation processes could be identified, denoted as peak A2 and peak B2. The redox-reaction mechanism is different than with glucose, which could be attributed to the first oxidation process appearing in peak A2, observed in the voltametric shape due to cleavage of organic-ketone groups, indicating that the formed products are different (mainly furanics), including hydroxymethylfurfural (HMF) and 2,5-furandicarboxylic acid (FDCA) (Kwon et al., 2015).

In the negative-going scan, the obtained current intensity in fructose (peak B2) is lower compared to glucose. At this point, sucrose oxidation is displayed in Figure 6, for **(c)** Pt/C and **(f)** Pt/CB respectively. As known, the sucrose molecule includes an aldehyde and ketone groups. Then, for this case, it could be seen the presence of three oxidation peaks, A3, B3 and C3, indicating three redox-reactions mechanisms. The peak A could be attributed to a first oxidation sequence of aldehyde group. In previous studies, some oxidation products have been identified, such as glucose, gluconic acid, glycolic acid and 5-Ketogluconic acid (Vedovato et al., 2020). According with the results, the peaks B3 and C3 presented lower current intensity, suggesting that the kinetics of electro-oxidation of sucrose is slower than glucose. These findings could be related with mass-transport limitations of the sucrose molecule, indicated by the diffusion coefficient which is lower than the glucose and fructose (Aroulmoji et al., 2012). As a manner of comparison, the voltametric features of **(g)** glucose, **(h)** fructose and **(i)** sucrose at concentration of 1 M were contrasted, see Figure 6. In glucose oxidation, a slightly higher current intensity is observed for Pt/CB at peaks A1 and C1. This effect was found for fructose oxidation, peak B2. This could be related to an enhanced adsorption and desorption of the organic molecule on the carbon black surface. For sucrose oxidation, the carbon black shows a slightly slower current intensity at peak C3, indicating that there is not a clear enhanced influenced by carbon black. This could also indicate that the oxidation processes or the organic compounds (as carbohydrates) depends strongly on the catalytic support, as well as the organic functional group of the molecule in turn.

**FIGURE 7:**
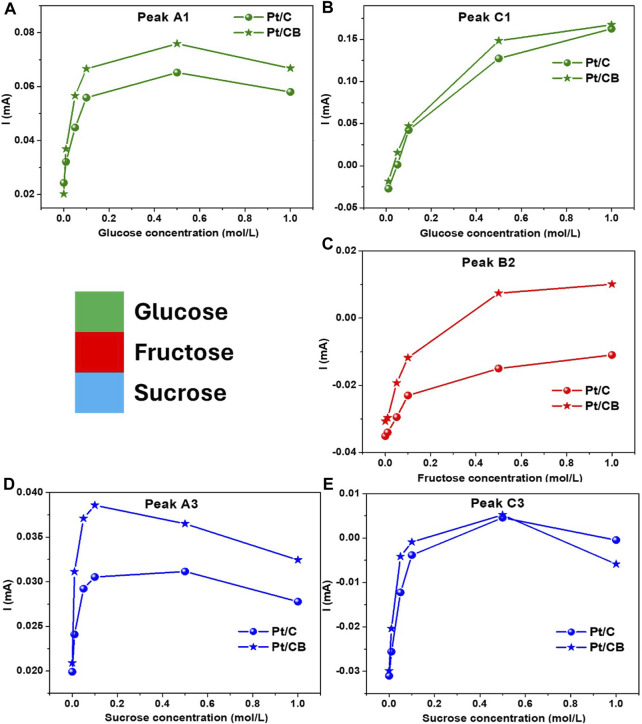
Profiles of current (mA) *versus* concentration (mol/L) of **(A, B)** glucose, **(C)** fructose and **(D, E)** sucrose for the catalysts in study.

In this context, the current-concentration plots are displayed in Figure 7 for all molecules in study. The profiles show the trend of the current with increasing concentration at the main redox peaks, Figure 7A–E. For example, at glucose-based solutions, Figure 7A,B, an increment for both oxidation process at peaks A1 and C1 can be observed. However, the redox interaction at A1 is higher at a concentration of 0.5 M, indicating that this process is limited to mass transport toward the electrode interface. For fructose, only the high-intensity current peak was taken, Figure 7C. For this case, the trend for the redox process at peak B2, increases as a function of concentration with lower magnitude as a slow kinetics oxidation of the ketone group in the fructose. Finally, sucrose oxidation current response was analyzed with respect to concentration. The peaks A3 and C3, Figure 7D, E respectively, shows a downward trend in both peaks, indicating that the redox process also depends on the mass-transport limitations towards the electrode surface.

**FIGURE 8:**
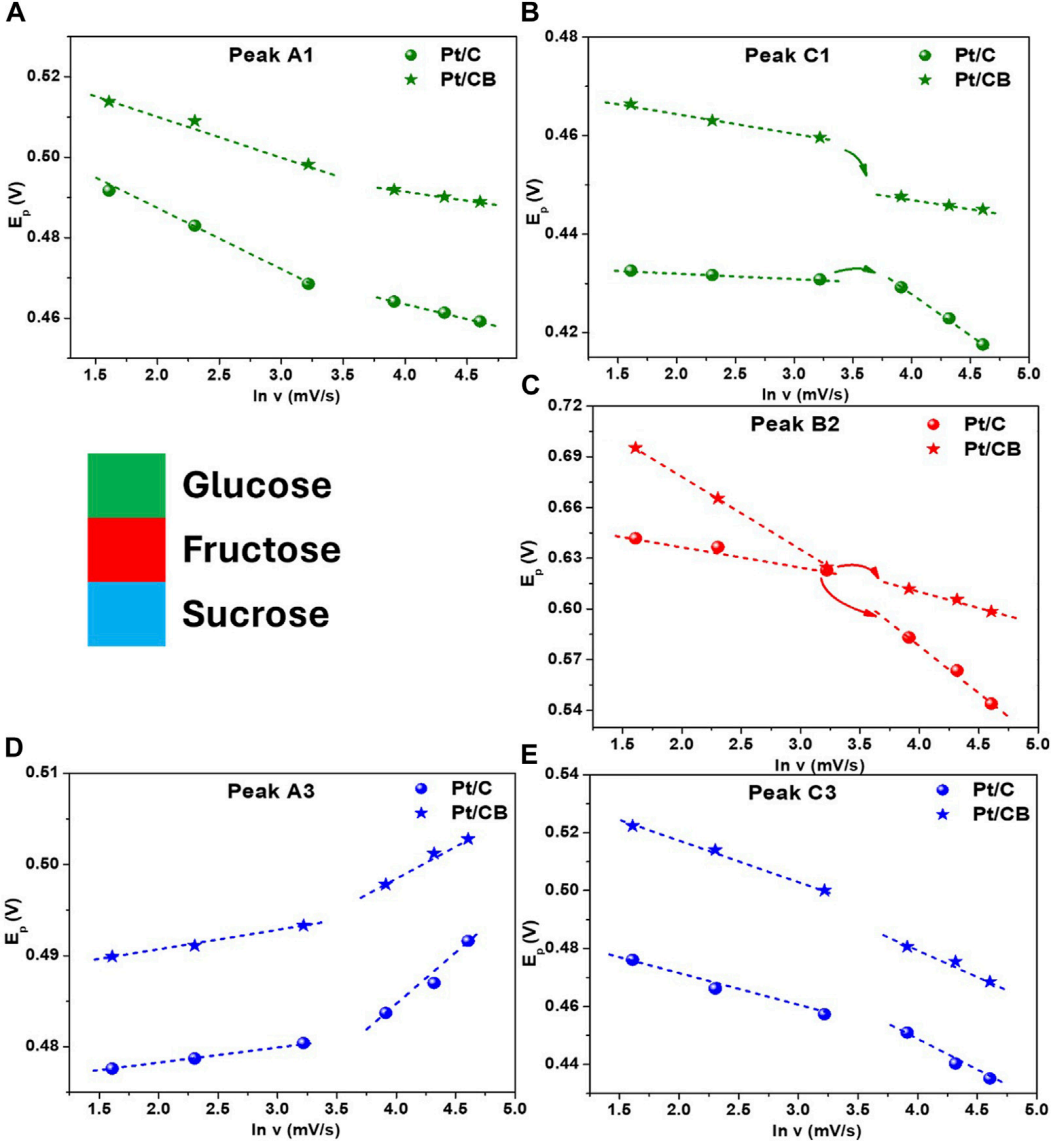
Profiles representing E_p_ *versus* ln v of **(A, B)** glucose, **(C)** fructose and **(D, E)** sucrose for the catalysts in study; from Figure 5, see text for details.

Following with this analysis, the peak potential (E_P_) *versus* scan rate (ln ν) for glucose **(a,b)**, fructose **(c)** and sucrose **(d, e)** is plotted in Figure 8. In all cases two well-defined slopes were observed at high and low scan rates, confirming that the carbohydrates-electro-oxidation is controlled by surface reactions. Furthermore, at high scan rates an increment of the irreversibility in the diffusion processes was observed (Elgrishi et al., 2018), being more marked for glucose and sucrose (peak A1 and A3 in Figure 8A,D respectively), corresponding to a first oxidation process and could be related with an aldehyde-organic group, included in both molecules. As a manner of comparison, Table 3 gives an overview of the reaction conditions and the obtained performance for Pt-based materials in the electro-catalytic oxidation of glucose in acid medium reported in literature (Skou, 1977; Luna et al., 1988; Luna et al., 1991; Kokoh et al., 1992; Popović et al., 1992; Watson and Attard, 2001; Kanninen and Kallio, 2018; Van der Ham et al., 2023).

**TABLE 3:** Summary of the performances reported in literature for electrooxidation of glucose in acid medium.

Electrode material	Electrolyte	Glucose concentration, M	Current peak, mA/cm^2^	Scan rate, mV/s	References
Pt/C, 10% wt Pt	0.5 M H_2_SO_4_	0.1	0.792	50	This work
Pt/CB, 10% wt Pt	0.5 M H_2_SO_4_	0.1	0.942	50	This work
PtPd, bulk alloy	0.1 M H_2_SO_4_	0.005	0.25	50	Watson and Attard (2001)
Pt, rotating electrode	1 M H_2_SO_4_	0.25	0.11	31	Skou (1977)
Pt/CNF, 5% wt Pt	0.1 M H_2_SO_4_	0.1	0.003	1	Van der Ham et al. (2023)
Pt, polycrystaline wire	0.5 M H_2_SO_4_	0.01	0.05	50	Luna et al. (1991)
Pt (111), wire monocrystaline	0.5 M H_2_SO_4_	0.01	0.15	10	Luna et al. (1988)
Pt (110), wire monocrystaline	0.5 M H_2_SO_4_	0.01	0.125	10	Luna et al. (1988)
Pt (100), wire monocrystaline	0.5 M H_2_SO_4_	0.01	0.110	10	Luna et al. (1988)
Pt/C, 60% wt Pt	0.1 M HClO_4_	0.1	0.018	20	Kanninen and Kallio (2018)
Pt grid	0.1 M HClO_4_	0.2	0.055	50	Kokoh et al. (1992)
Pt (111), single crystal	0.1 M HClO_4_	0.1	4.35	50	Popović et al. (1992)
Pt (100), single crystal	0.1 M HClO_4_	0.1	0.78	50	Popović et al. (1992)

#### 3.2.3 Mass-charge analysis using electrochemical impedance spectroscopy (EIS)

**FIGURE 9:**
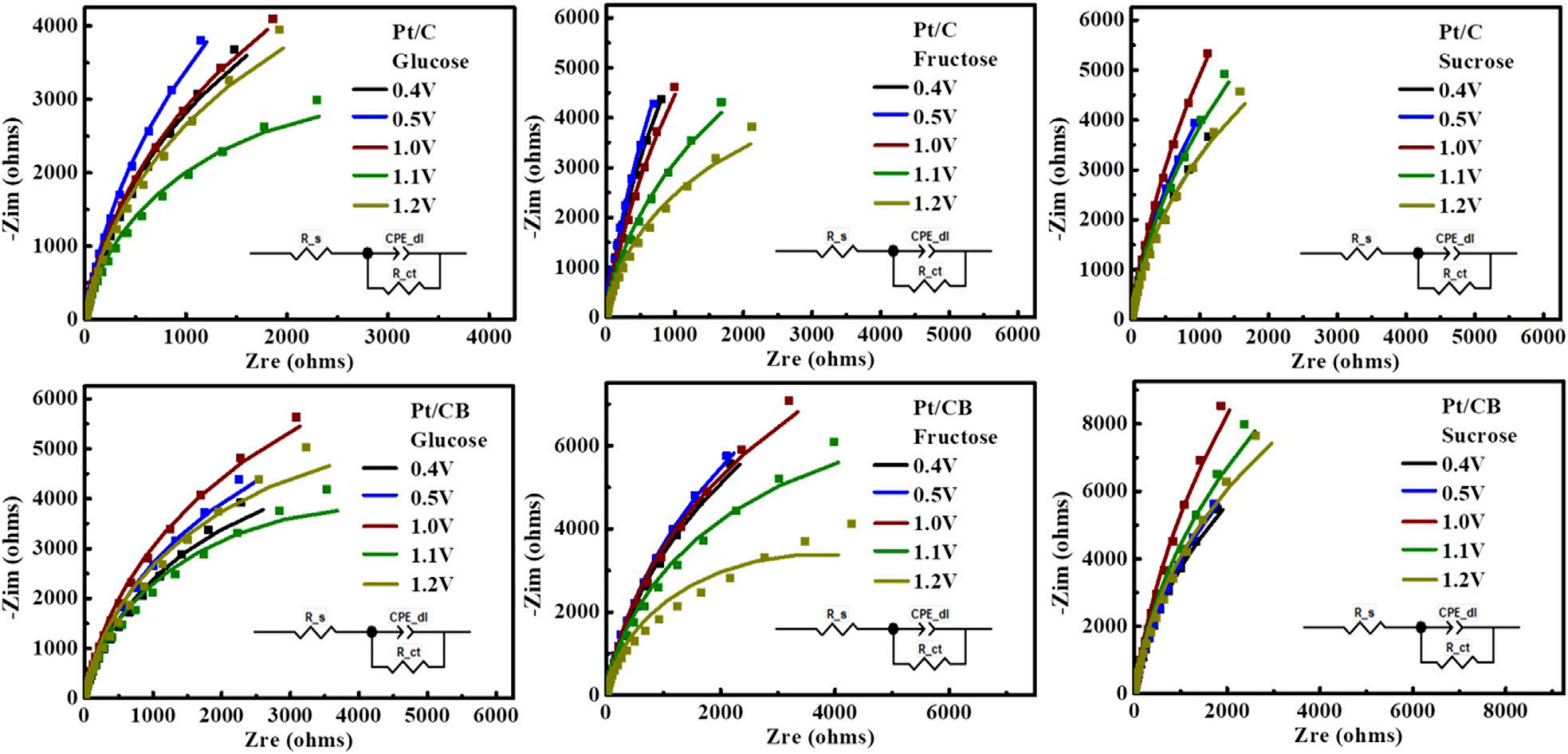
Nyquist plots corresponding to the Pt/C and Pt/CB catalysts in 0.5 M H_2_SO_4_ with 0.5M glucose, fructose, or sucrose solution I n 0.4,0.5,1.0,1.1,1.2 V vs. SHE. The inset shows the equivalent circuit used for fitting EIS data.

To further study mass-charge interactions at the electrode surface, electrochemical impedance spectroscopy (EIS) measurements were carried out. In EIS measurements a trend-interaction with respect to the catalytic support used was demonstrated. In Figure 9, the Nyquist plots illustrates the results for Pt/C and Pt/CB electrodes in a 0.5 M H_2_SO_4_ solution containing 0.5 M of the corresponding carbohydrate (glucose, fructose, or sucrose), at applied potential of 0.4, 0.5, 1.0, 1.1 and 1.2 V vs. SHE. The obtained Nyquist plots exhibit a semi-circle with a significant difference for Pt/C and Pt/CB. Analysis of the experimental data was performed through a fit with the corresponding equivalent circuit, as shown in the insets of Figure 9. The circuit parameters are denoted as follows: R_s is the solution resistance, CPE_dl-T represents the constant phase element linked to the double-layer capacitance, CPE-P represent rough surface electrode value between 0.9 and 1 and R_ct is the charge transfer resistance (Jorcin et al., 2006; Magar et al., 2021).

The electric parameters derived from the EIS experimental data for Pt/C and Pt/CB catalysts are presented in Table 4, Table 5. The solution resistance was found in the range of 11 Ω–19 Ω. In all evaluations, the capacitance (CPE_dl-T) revealed that the support of carbon black present a lower value than carbon Vulcan. In addition, lower magnitude of charge transfer resistance (R_ct) suggest two potential ranges: hydrogen adsorption and the oxidation of corresponding carbohydrates, verifying the results obtained from CV (Largeaud et al., 1995). For glucose, the oxidation process occurs at potential of ca. 0.4 V and 1.1 V vs. SHE. For fructose, the oxidation potential was observed at ca. 0.4 and 1.2 V vs. SHE; whereas for sucrose, it was found to be at ca. 0.4 and 1.2 V vs. SHE, elucidating-confirming the oxidation of these molecules.

**TABLE 4:** Electric parameters of equivalent circuit of Pt/C electrocatalysts of glucose, fructose and sucrose at different concentrations using 0.5 M H_2_SO_4_ in different potentials values.

V vs. SHE	R_s [Ω]	% Error	CPE_dl-T [μF]	% Error		CPE_dl-P	% Error	R_ct [Ω]	% Error
Glucose									
0.4	16.77	0.41	362.58	0.68		0.9431	0.22	12,273	4.40
0.5	16.69	0.40	374.90	0.66		0.9554	0.21	18,195	6.41
1.0	16.49	0.42	326.60	0.70		0.9406	0.22	13,238	4.42
1.1	16.43	0.45	332.11	0.78		0.9372	0.24	6,392	2.53
1.2	16.41	0.44	329.56	0.75		0.9429	0.23	10,822	3.88
Fructose									
0.4	14.84	0.41	350.05	0.66		0.9530	0.21	42,306	13.89
0.5	14.84	0.43	360.93	0.67		0.9518	0.21	57,036	19.62
1.0	14.75	0.43	331.89	0.66		0.9391	0.21	37,340	11.78
1.1	14.73	0.45	325.95	0.71		0.9440	0.22	15,018	5.01
1.2	14.68	0.50	329.25	0.81		0.9446	0.25	9,197	3.61
Sucrose									
0.4	16.63	0.36	390.82	0.61		0.9416	0.20	17,393	5.92
0.5	16.75	0.38	374.56	0.63		0.9427	0.20	28,782	9.64
1.0	17.34	0.39	281.26	0.62		0.9431	0.19	46,491	11.62
1.1	17.36	0.40	300.58	0.65		0.9411	0.21	25,674	7.20
1.2	17.34	0.43	314.60	0.70		0.9423	0.22	17,236	5.94

**TABLE 5:** Electric parameters of equivalent circuit of Pt/CB electrocatalysts of glucose, fructose and sucrose at different concentrations using 0.5 M H_2_SO_4_ in different potentials values.

V vs. SHE	R_s [Ω]	% Error		CPE_dl-T [μF]	% Error	CPE_dl-P	% Error	R_ct [Ω]	% Error
Glucose									
0.4	11.13	0.46		277.00	0.70	0.9124	0.20	10,143	2.95
0.5	11.16	0.39		269.30	0.59	0.9201	0.17	12,907	3.06
1.0	11.20	0.40		214.00	0.59	0.9307	0.16	15,599	2.93
1.1	11.17	0.51		211.20	0.78	0.9321	0.21	8,445	2.21
1.2	11.15	0.54		210.50	0.81	0.9375	0.23	11,094	2.90
Fructose									
0.4	18.57	0.29		237.72	0.47	0.9266	0.14	21,578	3.47
0.5	18.54	0.30		232.39	0.47	0.9272	0.14	25,152	3.98
1.0	18.48	0.33		184.15	0.52	0.9376	0.15	21,604	3.03
1.1	18.46	0.39		183.00	0.63	0.9443	0.18	13,419	2.34
1.2	18.41	0.64		189.58	1.08	0.9473	0.30	7,403	2.44
Sucrose									
0.4	14.88		0.25	253.98	0.40	0.9311	0.12	25,695	3.65
0.5	14.85		0.25	249.90	0.38	0.9315	0.11	29,457	4.02
1.0	14.75		0.27	174.78	0.40	0.9415	0.11	59,286	5.92
1.1	14.71		0.29	179.89	0.44	0.9412	0.12	36,115	4.07
1.2	14.71		0.33	181.31	0.50	0.9472	0.14	27,452	3.56

#### 3.2.4 *In-situ* analysis of reaction products

**FIGURE 10:**
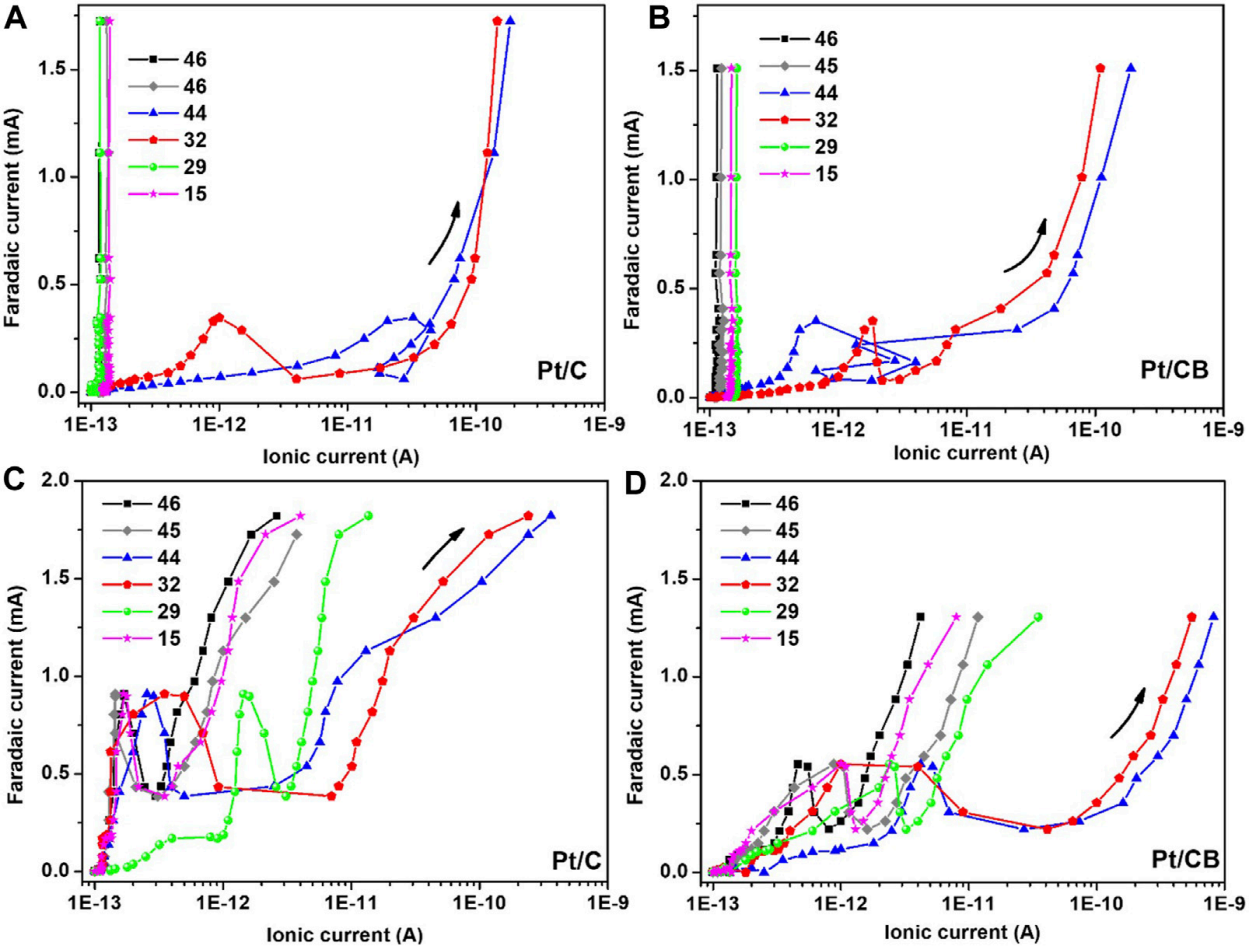
Faradic current vs ionic current profiles of the detected signals for **(A–C)** Pt/C and **(B–D)** Pt/CB in **(A, B)** absence and in **(C, D)** presence of glucose at concentration of 0.5 M. Data was extracted from datasets DEMS measurements.

The analysis of formed gaseous and/or volatile species from the glucose oxidation reaction (which showed more viability in the presented results from CV-EIS analysis) at the interface of Pt/C and Pt/CB during anodic polarization was carried out using a differential electrochemical mass spectrometry (DEMS) setup in 0.5 M H_2_SO_4_ solution in the absence and in the presence of the organic molecule in turn. Mass spectrometric signals corresponding to fragment-species such as [(HCOOH)-H^+^)] (m/z = 46), [HCOOH] (m/z = 45), CO_2_ (m/z = 44), O_2_ (m/z = 32), [CHO^+^] (m/z = 29) and [CH_3_^−^] (m/z = 15) was followed. The mass-signals m/z = 45 and 46 were assigned to formic acid, and m/z = 29 and 16, could be related to acetaldehyde, according with their corresponding mass-fragments (Morooka et al., 2008; Baasandorj et al., 2015). CV-faradic current and ionic current (IC) from DEMS are depicted in Figure 10A–D, for simplicity, only the positive-going scan is shown. In absence of glucose (see Figure 10A,B), generation of O_2_ and CO_2_ from mass-to-charge ratio (m/z) 32 and 44 is observed for both supports used.

Both materials (Pt/C and Pt/CB) show similar behavior concerning ionic current, with a high faradaic current at Pt/C. Conversely, in the presence of glucose (Figure 10C,D), the detected species show higher faradaic current at Pt/C. However, ionic current linked with acetaldehyde (m/z = 29, 15), molecular oxygen (m/z = 32) and CO_2_ (m/z = 44) indicated a higher value at the interface of platinum supported on CB as a major number of species are generated; also linked with the slope-variation with respect to the support employed. In addition, CO_2_ generated from this kind of experimental setup could be electrochemically reduced to produce added-value compounds such as methanol or methane (Mora-Hernandez et al., 2021b).

## 4 Conclusion

In this work, the electrooxidation of glucose, fructose and sucrose were investigated using Pt-nanoparticles supported on two different carbon-based materials: carbon Vulcan and carbon black. It was found that the catalytic activity in terms of faradic-current in the potential interval from 0.05 to 1.2 V vs. SHE was in the order Glucose > Sucrose > Fructose for both catalysts. For the oxidation reactions (mainly in glucose and fructose), higher currents were observed with carbon black-containing sample. This suggests that CB could modulated the kinetics of organic-molecules-adsorption-desorption and redox-process; putting in clear a notable difference between the supports as demonstrated by ECSA and capacitance calculations. On the other hand, during glucose oxidation, DEMS measurements clearly showed the trend of the IC-intensity associated with products as carbon dioxide, oxygen, acetaldehyde, and formic acid. In this context, the IC-curve shapes with glucose-containing solution displays substantial differences showing that there is a faster kinetics of products-generation at Pt-nanoparticles on carbon-black. According to this, the structural and morphological properties (such as crystal structure, crystallite size, degree of structural order and particles shape and distribution) obtained using XRD, SEM-EDS, Raman and XPS play a crucial role in the redox process for energy generation and/or value-added products from these kinds of organic molecules.

## Data Availability

The raw data supporting the conclusion of this article will be made available by the authors, without undue reservation.
